# *Acanthopsaron* Fricke, 2024 is a junior synonym of *Parapercis* Bleeker, 1863, and *A. striatum* Fricke, 2024 is a junior synonym of *P. fuscolineata* Fourmanoir, 1985 (family Pinguipedidae)

**DOI:** 10.3897/zookeys.1279.186008

**Published:** 2026-05-05

**Authors:** Hsuan-Ching Ho, Yo Su

**Affiliations:** 1 Department and Graduate Institution of Aquaculture, National Kaohsiung University of Science and Technology, Kaohsiung, Taiwan Department and Graduate Institution of Aquaculture, National Kaohsiung University of Science and Technology Kaohsiung Taiwan; 2 National Museum of Marine Biology and Aquarium, Pingtung, Taiwan International Doctoral Program of Marine Science and Technology, National Sun Yat-sen University Kaohsiung Taiwan; 3 Taiwan Ocean Research Institute, National Institutes of Applied Research, Kaohsiung, Taiwan National Museum of Marine Biology and Aquarium Pingtung Taiwan; 4 Australian Museum, Sydney, Australia Australian Museum Sydney Australia; 5 International Doctoral Program of Marine Science and Technology, National Sun Yat-sen University, Kaohsiung, Taiwan Taiwan Ocean Research Institute, National Institutes of Applied Research Kaohsiung Taiwan

**Keywords:** Biodiversity, Hemerocoetidae, ichthyology, invalid name, misidentification, synonymy

## Abstract

Fricke (2024) described a new genus and a new species, *Acanthopsaron striatum* Fricke, 2024, based on a single specimen. A re-examination of the holotype revealed several errors in the original description, including the numbers of dorsal- and anal-fin rays. Our results suggest that it is not a member of Hemerocoetidae and is in fact conspecific with *Parapercis fuscolineata* Fourmanoir, 1985 (family Pinguipedidae). A detailed observation of the holotype is provided and compared to data of other specimens of *P. fuscolineata*. As we found no difference between those two species, we propose that *Acanthopsaron* Fricke, 2024 is a junior synonym of *Parapercis* Bleeker, 1863, and *A. striatum* is a junior synonym of *P. fuscolineata*.

Fricke (2024) described a new genus based on *Acanthopsaron striatum* Fricke, 2024, a new species from New Caledonia based on a single, holotype specimen (MNHN 2024-0429, 44.2 mm SL; Fig. 1). The genus is monotypic. Examination of the holotype revealed that the specimen is actually a member of the genus *Parapercis* Bleeker, 1863, family Pinguipedidae. A detailed observation of the specimen is provided and compared to the potential conspecific species.

**Figure 1.**
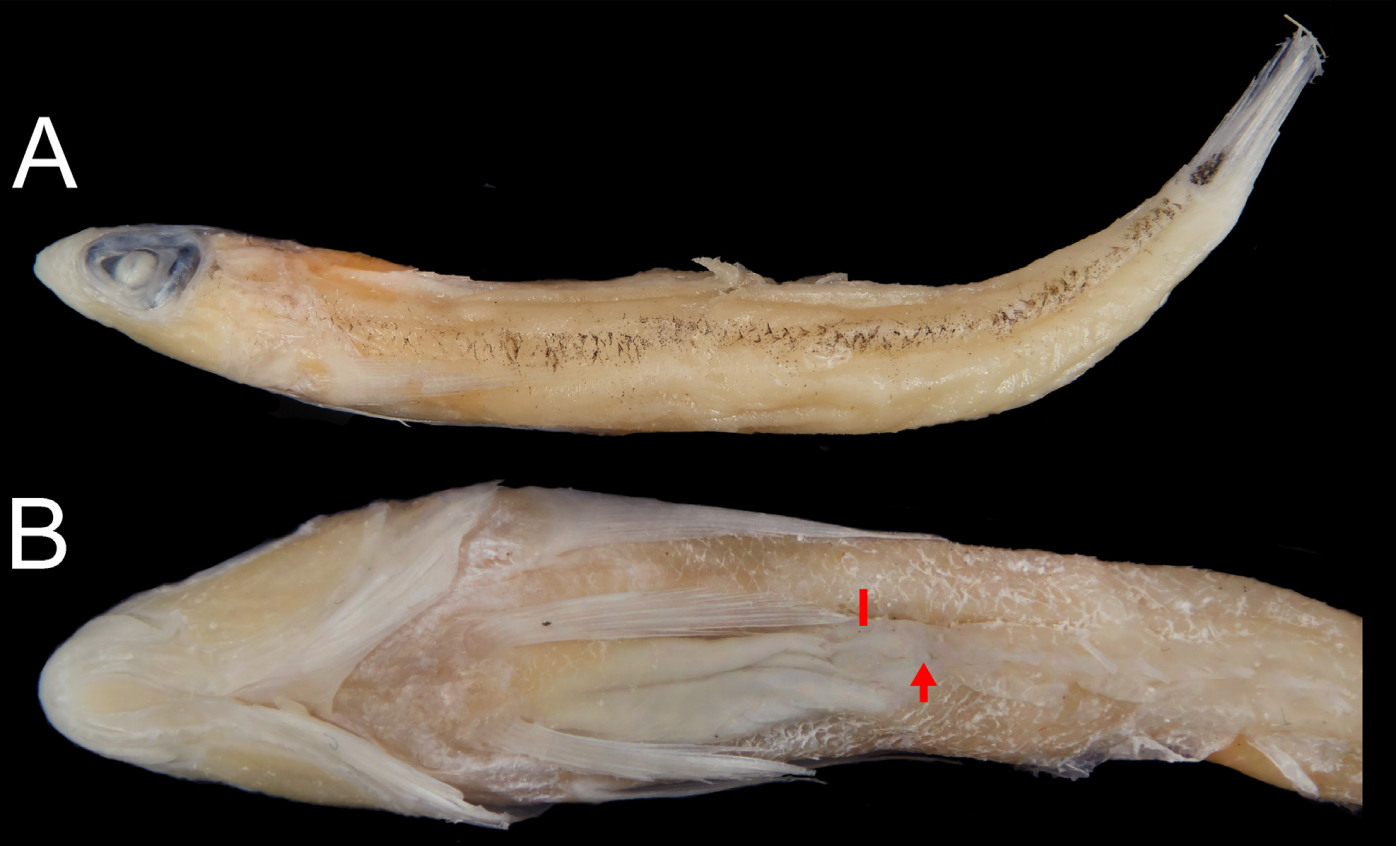
Holotype of *Acanthopsaron striatum*, MNHN 2024-0429, 44.2 mm SL. **A**. Lateral view; **B**. Close-up of ventral region, featuring genital papilla (arrowed) and the end of pelvic fin (bar).

Counts and measurements follow Randall et al. (2008). The specimen (holotype of *A. striatum*) examined in this study is deposited at Muséum national d’Histoire naturelle, Paris, France (**MNHN**). Comparative data were retrieved from Ho (2015) and Nakayama et al. (2016). Standard length is abbreviated as SL.

Our results reveal that the drawing and the data provided by Fricke (2024) does not correspond to the holotype in several critical ways. Six diagnostic characteristics—a single continuous dorsal fin with five spines; a single anal-fin spine; branched caudal-fin rays 13; two continuous rows of teeth the on vomer; iris flaps absent; lateral-line scales not serrated on posterior margin—serve to exclude the species from the family Hemerocoetidae, which has two distinctly separated dorsal fins, only soft rays on the anal fin, branched caudal-fin rays 7 or 8, vomerine teeth absent or divided into two patches, when present, iris flaps present in most species, and posterior lateral-line scales serrated (Nelson 2001; Odani and Imamura 2011; Nelson et al. 2016).

Table 1 summarizes the characteristics provided in the original description of *A. striatum*, compared to those observed directly from the holotype by us. The photograph of the specimen in fresh condition and the body size reported by Fricke (2024) confirm that the specimen examined here is the same one. However, many meristic values and morphological features are quite different, which leads us to question whether these are observations taken from the same individual.

**Table 1.** Summary of morphological characteristics provided in the original description of *Acanthopsaron striatum*, compared to our observations of the holotype.

Fricke 2024	Our observations (this study)
Dorsal-fin rays VII, ca 18, with the fifth spine longest; two dorsal fins	Dorsal-fin rays V, 23; their lengths progressively longer posteriorly; last spine largely connected to first ray by membrane
Anal-fin rays mostly branched. Anal-fin rays ca 23 in total	Anal-fin rays I, 19, mostly damaged now
Pectoral-fin rays 17 in total	Same
Branched caudal-fin rays 10. Caudal-fin rays 18 in total (including procurrent rays)	Branched caudal-fin rays 13 (7 + 6). Caudal-fin rays 9 + 8 = 17 (not including procurrent rays)
Scales small, cycloid. Lateral-line scales 60, peripheral ctenoid	Body scales small, lateral-line scales ca 60; remaining scales all ctenoid
Pelvic fin short, not reaching to anus when adpressed	Tip of the pelvic fin reaches the anterior margin of the anus, not reaching the genital papilla when adpressed (Fig. 1B)
Snout terminal, neither jaws projecting	Snout short, not projecting. Mouth terminal, with the lower jaw slightly included when mouth closed
Opercle with a single, strong spine. Subopercle with a weak spine	Opercle with a single strong spine. Subopercle with a single, short spine at angle
Scale rows above lateral line [sic!], below 6	Scale rows above lateral line ca 4, below ca 10
Predorsal scales 7	Not available
Gill rakers 2 + 7 = 9	Gill rakers 5 + 12 = 17
Maxillary tentacle, maxillary spine, barbel on lower-jaw tip, anterior-nostril projections, iris flap absent; maxillary notch present, weakly developed	Same

Table 2 lists selected characteristics that are consistent with those of *Parapercis fuscolineata* Fourmanoir, 1985 (type locality: the Philippines), provided by Ho (2015) and Nakayama et al. (2016). Given the observed morphological evidence, one will not hesitate to conclude that the holotype of *A. striatum* is referable to *P. fuscolineata*.

**Table 2.** Selected characteristics of *Acanthopsaron striatum* observed by the authors and compared to those of *Parapercis fuscolineata*.

Holotype of *A. striatum*; this study	*P. fuscolineata*; Ho 2015; Nakayama et al. 2016
Dorsal-fin elements V, 23	Dorsal-fin elements IV, 23 or V, 23
Anal-fin elements I, 19	Anal-fin elements I, 19
Pectoral-fin rays 17	Pectoral-fin rays 17–19
Lateral-line scales 60	Lateral-line scales 60–63
Scale rows above lateral line ca 4	Scale rows above lateral line 3.5–4.5
Scale rows below lateral line ca 10	Scale rows below lateral line 11–12
Gill rakers 5 + 12 = 17	Gill rakers 4–7 + 8–13 = 12–20
Pseudobranchial filaments 9	Pseudobranchial filaments 10–15
A broad, dark stripe along body and a large ocellus on upper base of caudle fin	A broad, dark stripe along body and a large ocellus on the upper base of caudle fin
A small black patch on the posterior portion of eye	A small black patch on the posterior portion of eye
Pelvic fin reaching the anterior margin of anus	Pelvic fin reaching slightly beyond anus
Two rows of continuous teeth on vomer; palatine teeth present (at least in 1 row)	Two rows of teeth on vomer and palatine
Front of lower jaw with 4 pairs of canines	Front of lower jaw with 4 pairs of canines
Scales mostly lost, remaining scales all ctenoid	Body mostly covered with ctenoid scales
Head length 25.8% SL	Head length 25.8–30.4% SL
Distribution: Grand Passage, New Caledonia, depth 277–292 m	Distribution: Japan, the Philippines, Vanuatu, New Caledonia, and Solomon Islands, depths from 180–355 m

In summary, our detailed examination of the holotype of *A. striatum* disagrees in several critical ways with the original description of Fricke et al. (2024). Based on the newly observed data and morphological features, we recognized the holotype of *A. striatum* as a member of Pinguipedidae, and agrees well with the original description and additional specimens of *P. fuscolineata*, which led us to conclude that the former genus as a junior synonym of *Parapercis* and the species as a junior synonym of *P. fuscolineata*.

## Additional information

### Use of AI

No use of AI was reported.

### Author ORCIDs

Hsuan-Ching Ho https://orcid.org/0000-0003-1154-601X

Yo Su https://orcid.org/0000-0002-3576-9229

### Data availability

All of the data that support the findings of this study are available in the main text.
